# A cross sectional study of requests for knee radiographs from primary care

**DOI:** 10.1186/1471-2474-8-77

**Published:** 2007-08-06

**Authors:** John Bedson, Kelvin P Jordan, Peter R Croft

**Affiliations:** 1 Primary Care Musculoskeletal Research Centre, Keele University, Staffordshire, ST5 5BG, UK

## Abstract

**Background:**

Knee pain is the commonest pain complaint amongst older adults in general practice. General Practitioners (GPs) may use x rays when managing knee pain, but little information exists regarding this process. Our objectives, therefore, were to describe the information GPs provide when ordering knee radiographs in older people, to assess the association between a clinical diagnosis of osteoarthritis (OA) and the presence of radiographic knee OA, and to investigate the clinical content of the corresponding radiologists' report.

**Methods:**

A cross sectional study of GP requests for knee radiographs and their matched radiologists' reports from a local radiology department. Cases, aged over 40, were identified during an 11-week period. The clinical content of the GPs' requests and radiologists' reports was analysed. Associations of radiologists' reporting of i) osteoarthritis, ii) degenerative disease and iii) individual radiographic features of OA, with patient characteristics and clinical details on the GPs' requests, were assessed.

**Results:**

The study identified 136 cases with x ray requests from 79 GPs and 11 reporting radiologists. OA was identified clinically in 19 (14%) of the requests, and queried in another 31 (23%). The main clinical descriptor was pain in 119 cases (88%). Radiologists' reported OA in 22% of cases, and the features of OA were mentioned in 63%. Variation in reporting existed between radiologists. The commonest description was joint space narrowing in 52 reports (38%). There was an apparent although non significant increase in the reporting of knee OA when the GP had diagnosed or queried it (OR 1.95; 95% CI 0.76, 5.00).

**Conclusion:**

The features of radiographic OA are commonly reported in those patients over 40 whom GPs send for x ray. If OA is clinically suspected, radiologists appear to be more likely to report its presence. Further research into alternative models of referral and reporting might identify a more appropriate imaging policy in knee disorders for primary care.

## Background

Knee pain affects an estimated 25–37% of people over 50 years [1-4] and is the commonest pain complaint amongst older adults in general practice [5]. Osteoarthritis is the most frequent diagnosis associated with this symptom but general practitioners can make this diagnosis either as the clinical syndrome (pain, stiffness, restricted movement) or on the basis of radiographic appearances (joint space narrowing, bony sclerosis, osteophytes). However, clinical osteoarthritis is not necessarily equivalent to radiographic disease since those aged over 45 with knee pain will have x ray changes consistent with osteoarthritis in 36–50% of cases [6-10], whilst between 24 – 56% of patients with radiographic knee osteoarthritis experience pain [6,11-18] We have previously investigated the influence of x rays on GPs' choice of care, and shown that, in identical clinical situations, x ray evidence of radiographic osteoarthritis altered their management of knee symptoms – even if the GP would not have chosen to x ray the knees in that situation [19]. We have now carried out a study to determine the content of information provided by GPs when they request a knee x ray, particularly whether they mention a clinical diagnosis of osteoarthritis (OA), and to estimate the association between a GP's clinical diagnosis of OA on an x ray request and the presence of radiographic knee OA on the radiologist's report.

Roland and van Tulder have suggested that, when reporting x rays of the back, radiologists should use pre-set statements which indicate that the appearance of 'spinal degeneration' may not be related to the patient's symptoms [20]. This is because in back pain, as with knee pain, radiographic findings do not necessarily equate with the clinical picture and, moreover, such statements might help to nullify anxiety and inappropriate restriction of activity which might arise from a diagnosis of degeneration. A further objective of our study was to investigate whether a similar problem might arise with knee x ray reports by determining the language and comments on severity contained in radiologists' reports on knee x rays.

## Methods

This was a cross sectional study of patients aged over 40, referred for an x ray of one or both knees to a district general hospital by their general practitioner. The study took place during an 11-week period commencing 14/4/2002 in one hospital in the West Midlands region of England, and involved prospective data collection of new x ray requests The hospital serves a population of approximately 370,000 patients and 190 GPs. A large proportion of the GPs in North Staffordshire would use this hospital as their chosen diagnostic centre for radiology referrals. In the UK system, all requests for knee radiography are made via GPs using standard referral forms. The GP radiology request form itself has two sections. The first asks for a free text entry of the clinical details which might either include a description of the symptoms or a proposed diagnosis. This is completed by the GP. The second part asks which radiograph the GP wishes to be performed.

The local research ethics committee approved the study. The study period was determined by practical issues relating to the radiology department's wish to limit the study period to 11–12 weeks and the availability of the senior radiographer for identification and collation of x ray requests and reports.

The attending radiographers identified all patients referred for knee x ray during the study period. The superintendent radiographer then obtained and copied the GP request card, removing the names of the patient but preserving the date, age and gender of the patient and clinical details. The GPs were subsequently allocated an individual code by the authors and their names were removed to allow us to anonymously identify the numbers of GPs requesting knee x rays, and how many in total each GP requested. The superintendent then copied the matching radiologist's report. The radiologist's name was removed from each report and replaced with a code number which had been allocated to that radiologist. This enabled an anonymised study of variation in the reporting between radiologists to be carried out. Each patient was given an identification code which was attached to both request and report card in order to allow the anonymised comparison of the items from the two cards.

Data was collected in two parts. Part one consisted of socio-demographic information and clinical details from the GP's request card. Part two concerned the radiologist's code number, their descriptive report of the x ray and any additional clinical information which they offered. A data extraction protocol was developed, covering in precise detail the items of information to be recorded in each case (this protocol is available on request from the authors). From the GP request form, 35 potential items of information were identified, and from the radiology report there were 31 possible items. The protocol was initially piloted by the two medical professionals authoring the study to ensure clear comprehension and repeatability. Subsequently, three separate health care professionals, all doctors, analysed each request and report, blinded to each other's findings and to the code number of the reporting radiologists. Discrepancies were resolved by consensus of two of the authors.

### Statistical Analysis

The percentage of patients for whom the referring general practitioner queried or diagnosed OA on the request form was calculated with a 95% confidence interval, as were the percentages of patients with clinical signs, symptoms and other details mentioned. Odds ratios with 95% confidence intervals were used to assess the association between GP OA diagnosis and the findings in the radiologists' reports. The radiologists' reports were grouped cumulatively in a hierarchical fashion according to the x ray terms used in the reports, and definitions of each group are described in Table 1.

**Table 1:** Definitions of the hierarchical grouping of radiological features from the radiologists' reports

Group Definition	Description
Group 1 Definition	The term 'osteoarthritis' or 'OA' is used.
Group 2 Definition	Those in group 1 plus those with the term 'degenerative disease'.
Group 3 Definition	Those in groups 1 and 2 plus those with any mention of specific radiographic features of osteoarthritis i.e. joint space narrowing, osteophytes, lipping, sclerosis or articular surface changes.

The associations between a GP's diagnosis or possible diagnosis of OA and the age and gender of the patient were assessed by odds ratios with 95% confidence intervals using multiple logistic regression. The influence on radiologists' reporting (based on the various groups defined in Table 1), of (i) the age/gender of the patient and (ii) whether the GP had queried or diagnosed OA, was assessed using multiple logistic regression. Due to the small numbers of x rays reported on by some of the radiologists, we identified the two radiologists reporting on the majority of x rays and grouped the rest together; we then assessed differences in reporting style by comparing each of the high reporters with the "rest".

Analysis was performed using SPSS 11.0 for Windows [21].

## Results

During the 11-week study period, 136 subjects were identified, of whom 74 (54%) were female. The mean age was 60.1 years (SD 10.7) and median 59 (range 40 – 85). A total of 79 GPs requested the x rays and the maximum number of x rays requested by any single GP was 5. There were eleven reporting radiologists with a median of 7 reports per radiologist (range 1 – 40). One radiologist (radiologist 7) reported on 40 x rays, a second (radiologist 8) on 39. The remainder each reported on 15 or less.

### GP request cards

Table 2 shows the information given by general practitioners on their request cards. Only two cards had no information at all about possible diagnosis or details of symptoms and signs. In total, 37% (95% CI 29%, 45%) of request cards had mention of either a possible diagnosis (*n *= 31) or diagnosis (*n *= 19) of knee OA. GPs offered details of clinical symptoms in 120 subjects (88%; 95% CI 82%, 93%), with pain the most common feature (119, 88%). The next most common symptoms were soft tissue swelling (15%), symptom duration (15%), and the site of symptoms (10%).

**Table 2:** Information and requests on GP request forms (*n *= 136)

Examine right knee	47 (35%)
Examine left knee	56 (41%)
Examine both knees	31 (23%)
Not stated	2 (1%)
Specific diagnosis of OA	19 (14%)
Possible diagnosis of OA	31 (23%)
Query other form of arthritis	2 (1%)
Mention of other diagnosis	8 (6%)
Clinical signs described	28 (21%)
Crepitus	11 (8%)
Joint tenderness	8 (6%)
Quadriceps muscle wasting	0 (0%)
Joint enlargement	1 (<1%)
Movement limitation	3 (2%)
Temperature or heat	3 (2%)
Effusion	11 (8%)
Clinical symptoms described	120 (88%)
Pain	119 (88%)
Joint stiffness	11 (8%)
Walking distance	0 (0%)
Knee locking	9 (7%)
Soft tissue swelling	21 (15%)
Knee instability	4 (3%)
Clicking	0 (0%)
Duration of symptoms	20 (15%)
Severity of symptoms	1 (<1%)
Incapacity state determinable	0 (0%)
Site of symptoms	14 (10%)
Request to grade severity	3 (2%)
Request to identify loose bodies	5 (4%)
Previous x ray mentioned	2 (1%)
Previous orthopaedic consultation or operation mentioned	8 (6%)
Treatment mentioned	3 (2%)
Medication	1 (<1%)
Physiotherapy	1 (<1%)
Steroid injection	1 (<1%)
Referral to orthopaedics or TKR suggested	1 (<1%)
Trauma mentioned	
Yes, trauma	12 (9%)
Yes, no trauma	6 (4%)
Social history given	2 (1%)
x ray view specified	6 (4%)

Clinical signs were described in 28 subjects (21%; 95% CI 15%, 28%), and most commonly this was either an effusion or crepitus (both for 11 (8%) patients). Combinations of different items of information occurred infrequently. Only 8 cards referred to osteoarthritis as well as detailing signs and symptoms. Patients with a sign reported by their GP were less likely to have a symptom reported, and vice versa (Chi-square test, *p *= 0.01). There was however no relationship between the use of signs or symptoms on the request form and the mention or possible diagnosis of knee OA.

Other aspects were mentioned rarely – for example in two (1%) subjects a social history was briefly detailed, treatment strategies were described in three (2%) subjects, and intention to refer to an orthopaedic surgeon in one subject alone. Requests which might relate to specific strategies of treatment were also unusual – for example a request to grade severity occurred in only three (2%) cases. Specification of x ray view (antero-posterior or lateral) appeared rarely (*n *= 6, 4%).

With respect to the GPs' request cards, table 3 looks at the associations between the GPs' mention of OA as a possible diagnosis on the request card and the gender and age of the patient. GPs appeared more likely to mention OA if the patient was female (OR 1.51; 95% CI 0.73, 3.11, *p *= 0.27), or if the patient was aged over 60 (OR 2.32; 95% CI 1.13, 4.77, *p *= 0.02).

**Table 3:** Characteristics associated with mention of OA diagnosis on GP request

*n *= 136	Total	Possible or specific OA diagnosis on GP request	
		*n *(%)	OR^a ^(95% CI)
Male	62	20 (32%)	1.00
Female	74	30 (41%)	1.51 (0.73, 3.11)
Age			
under 60	69	19 (28%)	1.00
60 & over	67	31 (46%)	2.32 (1.13, 4.77)

^a ^adjusted for other presented variable

### Radiologists reports

The reported prevalence of radiographic OA in this study population (group 1 definition) shown in table 4, was 22% (95% CI 16%, 30%). The prevalence of features of radiographic OA (group 3 definition) was 63% (95% CI 54%, 70%).

**Table 4:** Information on radiologist reports – TFJ and PFJ combined (*n *= 136)

OA in 1 or both joints	30 (22%)
Not OA/Normal	50 (37%)
No description	56 (41%)
OA grade (*n *= 30 – most severe taken)	
Mild	18
Moderate	7
Severe	3
Indeterminate	2
Degenerative change in 1 or both joints	35 (26%)
Mild	16 (12%)
Moderate	8 (6%)
Severe	5 (4%)
Indeterminate	6 (4%)
Joint space grade descriptions	52 (38%)
Mild	26 (19%)
Moderate	9 (7%)
Severe	7 (5%)
Indeterminate	10 (7%)
Osteophyte grade descriptions	11 (8%)
Mild	5 (4%)
Moderate	1 (<1%)
Indeterminate	5 (4%)
Lipping/spiking/pointing grade	33 (24%)
Mild	22 (16%)
Moderate	2 (1%)
Severe	2 (1%)
Indeterminate	7 (5%)
Scelorosis grade – Indeterminate	2 (1%)
Articular surface grade	4 (3%)
Severe	1 (<1%)
Indeterminate	3 (2%)
Everything else normal	27 (20%)
Referral or other management suggested	3 (2%)

Table 4 also shows the frequency with which radiologists reported individual abnormalities. The commonest description was of joint space narrowing, 52 reports (38%; 95% CI 31%, 47%), followed by lipping, 33 reports (24%; 95% CI 18%, 32%). The radiologists graded the severity of radiographic OA, when they reported it, in 28 of the reports (93% of OA reports), 18 (60% of OA reports) being described as mild and 3 (10%) as severe. Only 12 (9%) reports had a grading of severe for one or more features of radiographic OA (group 3 definition). A course of action was only recommended in 2% of all patients.

Table 5 examines the association between a GP's diagnosis or possible diagnosis of OA on the request card, and the radiologist's reporting of OA or radiographic features of OA. After adjusting for the age and gender of the patient, and the reporting radiologist, there was an apparent although non significant increase in the reporting of knee OA when the GP had diagnosed or queried it (OR 1.95; 95% CI 0.76, 5.00, *p *= 0.17). This association disappeared when considering any feature of radiographic OA (group 3 definition) mentioned in the x ray report (OR 1.05; 95% CI 0.48, 2.29, *p *= 0.90). Radiologists reported a feature and not specifically OA or degenerative disease in 25 patients, 7 (28%) of which the GP considered to have OA. Radiologists tended to be less likely to report only features if the GP mentioned or queried the presence of OA (OR 0.48; 95% CI 0.17, 1.35, *p *= 0.17).

**Table 5:** Associations with (i) the term 'osteoarthritis' (group 1 definition) (ii) the terms 'osteoarthritis' and 'degenerative joint disease' (group 2 definition) and (iii) the terms 'osteoarthritis', 'degenerative joint disease' and any descriptive radiological feature of knee osteoarthritis (group 3 definition), from Radiologists' reports, summarised by the odds ratio (95% confidence interval)

*n *= 136	Total	Group 1 Definition		Group 2 Definition		Group 3 Definition	
		*n *(%)	OR^a ^(95% CI)	*n *(%)	OR^a ^(95% CI)	*n *(%)	OR^a ^(95% CI)
Male	62	16 (26%)	1.00	33 (53%)	1.00	43 (69%)	1.00
Female	74	14 (19%)	0.55 (0.22, 1.40)	27 (36%)	0.48 (0.24, 0.99)	42 (57%)	0.56 (0.27, 1.17)
Age under 60	69	13 (19%)	1.00	24 (35%)	1.00	37 (54%)	1.00
60 & over	67	17 (25%)	1.40 (0.55, 3.56)	36 (54%)	2.05 (1.00, 4.23)	48 (72%)	2.13 (1.01, 4.49)
Radiologists							
Others	57	7 (12%)	1.00	21 (37%)	1.00	38 (67%)	1.00
Code 7	40	20 (50%)	7.54 (2.69, 21.17)	21 (53%)	1.96 (0.83, 4.61)	28 (70%)	1.17 (0.48, 2.86)
Code 8	39	3 (8%)	0.62 (0.15, 2.59)	18 (46%)	1.60 (0.67, 3.81)	19 (49%)	0.46 (0.19, 1.09)
GP diagnosis/possible diagnosis of OA							
No	86	15 (17%)	1.00	34 (40%)	1.00	52 (60%)	1.00
Yes	50	15 (30%)	1.95 (0,76, 5.00)	26 (52%)	1.60 (0.75, 3.40)	33 (66%)	1.05 (0.48, 2.29)

^a ^adjusted for other presented variables

Table 5 shows that females were less likely to have a report of OA or degenerative disease by the radiologist (group 2 definition, OR 0.48: 95% CI 0.24, 0.99, *p *= 0.047) or any feature of radiographic OA (group 3 definition, OR 0.56, 95% CI 0.27, 1.17, *p *= 0.12). There was also a significant association between age over 60 and the reporting of the presence of OA or of OA features (group 3 definition, OR 2.13; 95% CI 1.01, 4.49, *p *= 0.046).

Table 5 also assesses the variation between the radiologists in their use of terms and descriptions. Radiologist 7 was significantly more likely to diagnose OA in the report than the other radiologists (OR 7.54; 95% CI 2.69, 21.17, *p *< 0.001). Although the associations were non significant, radiologist 8 appeared less likely to diagnose OA (OR 0.62; 95% CI 0.15, 2.59, *p *= 0.51) and more likely to use the term "degenerative disease" than the other radiologists (group 2 definition OR 1.60; 95% CI 0.67, 3.81, *p *= 0.29). Overall, radiologist 8 was less likely to use any descriptions of individual features of radiographic OA (group 3 definition, OR 0.46; 95% CI 0.19, 1.09, *p *= 0.08).

## Discussions and conclusion

In a series of 136 requests for x rays of the knee in older people, there was clear variation in the information provided by the GPs about their diagnostic decision. In only 14% was a specific diagnosis of OA was recorded on the request form, whilst a possible diagnosis of OA was recorded in a further 23%.

The overall prevalence of radiographic knee OA reported by the radiologists was 22%, and when combined with referrals described as having one or more radiographic features of knee OA, the prevalence was 63%. Previous population studies in those aged over 45 with knee pain have found x ray changes consistent with osteoarthritis in 36–50% of cases [6-10]. Radiologists graded the severity of OA in 93% of patients with OA. Only 9% of patientsin this consecutive series of referrals by GPs for radiography were identified as having OA of a severity which might be expected to influence management choices. What is unknown is the absolute level of radiographic osteoarthritis if it were judged using standard repeatable methods of assessment. However this was not the point of the study since in clinical practice the standard with which clinicians operate is the opinion and judgement of the radiologist. We were therefore concerned in this study with the comparison of the words and phrases used by radiologists and the requesting GPs.

All radiologists in the reporting department and all GPs in the referring practices of North Staffordshire were informed of the nature of the study well in advance of the data collection. However they were not informed of the study period during which the requests and reports were to be reviewed. Although some 'Hawthorne' effect might have been created because of awareness of an imminent study, we considered that the lack of awareness of the data collection period offered some protection against this, and that the focus of the analysis on internal comparisons means that any effect of foreknowledge was unlikely to have been a bias on study results. As the length of the study period was limited by practical issues, this also limited the number of requests included in the study. As a result, although the estimated size of the main associations with reporting found here (measured by odds ratios) were high, they were sometimes non significant at the conventional 5% level.

Our findings reflect activity in one central outpatient x ray department in the West Midlands of England, and this may not reflect the entire cross-section of referrals and reporting that occurs within the United Kingdom. Another potential limitation to the study is that the majority of the reports were completed by two radiologists. However, since they reported in very different ways, and the other radiologists occupied a spectrum between them, our results at least indicate the likely range of reporting that might be encountered in UK radiology departments. It also highlights the variation between individual radiologists in the information they report. A previous study by Naik found variations in reporting amongst radiologist which appeared to be individually consistent in other modalities such as ultrasound, but whether this extends to plain radiography is unknown [22].

Unlike the radiologists, the GPs' requests were not analysed for variation in clinical content between GPs. By identifying each GP with a code however it was possible to quantify the number of GPs requesting knee radiographs, and how many knee x rays individual GPs requested during the study period. In total 79 GPs requested x rays, with 5 being the maximum number of knee x rays requested by any one GP. As a consequence of this we can be confident that our findings are not due to a 'clustering' effect generated by one or two individual GPs requesting large volumes of knee x rays. In addition, our estimate of the prevalence of different characteristics might reflect such issues as the study period, but this should not have affected the internal study comparisons that we were examining.

Patients in this study represent a group whom GPs have elected to x ray as a way of investigating knee disorders that present to general practice. Of these, 63% were found to have features of radiographic osteoarthritis. This appears to be higher than previously described in several population-based studies in people aged over 45 with knee pain where x ray changes consistent with osteoarthritis were found in 36–50% of cases [6-10]. This difference is likely to reflect patient self-selection when attending general practice, and GP selection of patients to x ray. Why GPs select patients to x ray appears to be multifactorial. A previous study has shown that the presence or absence of clinical OA does not influence the decision, and that it is more likely to be related to an individual's propensity to use x rays or not in the first place [19]. Differential selection of patients for referral to x ray in this study is suggested by the age and gender characteristics which link with the attribution of OA on the forms. Older age was associated with the GP mentioning a possible diagnosis of OA on the request form, and with radiographic OA on the report forms. This fits with the demographic characteristics of OA [23]. A possible diagnosis of OA was associated with female gender of the patient on the request form, but radiographic OA was more likely to be found in men, the opposite of epidemiological studies which have indicated it is more common in women [2,23], thus suggesting that gender is differentially influencing the GPs' diagnostic and referral pattern.

Of those patients whom GPs' considered might have OA, two-thirds had either a radiographic diagnosis or description of OA features. However, 60% of those who did not have any mention of OA on the request card also had a diagnosis or description of OA features on the radiographic report. The conclusion is that the majority of older patients referred with knee pain will have radiographic evidence of OA features detailed by the radiologist. This again is likely to reflect patient self-selection and subsequent GP choice to x ray since population studies have only found up to 50% of patients with knee pain have x ray changes of OA [6-10]. Given our previous findings that x ray features affect management regardless of the clinical picture [19], then it seems likely that this levels of radiographic reporting may influence treatment.

Yet there is clearly variation in radiographic certainty. OA appeared more likely to be specifically diagnosed if the GP mentioned it on the request form. Morgan found that GPs' would x ray knees in 42% of cases to confirm or assess degenerative change whilst 40% had no clear diagnosis in mind [24]. However, it is unknown whether this has any influence over the actual information presented on the x ray request card. In this study if it was not mentioned on the request form, there was an increased likelihood of the radiologist only reporting individual radiographic features. Either the radiologists' reporting language is affected by the GP's certainty, or those patients whom the GP consider may have OA are clinically and radiographically in a more "certain" group. However, most variation in the use of terms occurs between radiologists. Whether this is important or not in terms of the influence on management cannot be answered by the current study, but as has previously been noted, radiologists will report across a wide spectrum of imaging modalities, including unenhanced radiography, in an individually consistent way [22]. In our study, each style of reporting informs the GP of the radiographic features, but the difference illustrates the lack of standardisation for the language and structure in reporting of knee x rays. This has potential implications since, as appears to be the case with the spine, unqualified reports of degenerative change, or the use of diagnostic terms such as arthritis, may affect a patient's response to their symptoms or create anxiety [20]. Radiologists may need to review their terminology and include statements to point out that the findings on the x ray may not account for the knee problem, and then GPs could convey this message positively to their patients.

GPs appear to offer relatively little information to the radiologists in their requests apart from the presence of pain (88%), and some clinical symptoms such as crepitus (8%). Rarely is a social history or details of current management included. Requests to radiologists for support in decision-making occur in fewer than 1% of cases. Apart from Morgan's study of requests for knee x rays in primary care which only looked at the personal reasons of GP's to refer, there is no other evidence in the literature to compare this finding with [24]. So the information that the radiologist generally receives consists of a description of pain and, in a third of cases, a potential diagnosis of knee OA. GPs may not always supply information in their requests because they want the definitive descriptive report supplied by the radiologist to help them to make a decision.

GPs did not often request help in management, and radiologists rarely suggested treatment options. Radiologists may choose not to offer advice on management because the presence of radiographic degenerative joint disease does not necessarily equate with the symptoms a patient may be experiencing [6,15,25,26]. Since radiologists receive relatively little clinical information in the x ray request, they might see it as inappropriate to suggest a course of action, in a patient with whom the radiologist is unfamiliar, or to base any advice on an x ray which might not reflect the clinical picture. In addition, radiologists will be familiar with current guidelines, such as the New Zealand criteria for referral for consideration of knee arthroplasty, where degenerative x ray changes rate relatively low as a factor in the decision to refer [27]. Offering such advice based purely on the x ray would be out of step with this.

In conclusion, GPs receive detailed reports from radiologists which, in nearly two-thirds of the subjects, describe degenerative joint disease. Radiologists report these x rays in general with a limited set of clinical details apart from pain and, in a third of cases, a suggestion from the GP that the patient might have knee OA. The content of the request and the report might be considered disparate phenomena in that one does not necessarily reflect or depend upon the other, except for the basic fact that the request card itself triggers the x ray. The practical issue is that if GPs act on the pathological findings, the x ray results might influence clinical outcome, and in particular such x ray findings may have a negative effect by diverting attention from treatment of symptoms and disability to management plans dictated exclusively by an x ray report which may or may not be relevant to the patient's actual problems. With the advent of the patient electronic record, accessible to all practitioners when dealing with an individual, the radiologist and GP may be able in the future to supply and access each other's information. This may lead to GPs only having to request the knee x ray and no more. Further research into such new models for referral and reporting the primary-secondary care interface might identify a more appropriate imaging policy in knee disorders.

## Competing interests

The author(s) declare that they have no competing interests.

## Authors' contributions

JB and PC conceived and designed the study. KJ was primarily responsible for statistical analysis. All authors contributed to the interpretation and writing of the paper, with prime responsibility taken by JB.

## Pre-publication history

The pre-publication history for this paper can be accessed here:


